# Influence of Adiposity on the Gut Microbiota Composition of Arab Women: A Case-Control Study

**DOI:** 10.3390/biology11111586

**Published:** 2022-10-28

**Authors:** Esra’a A. Aljazairy, Sara Al-Musharaf, Manal Abudawood, Basmah Almaarik, Syed D. Hussain, Abdullah M. Alnaami, Shaun Sabico, Nasser M. Al-Daghri, Mario Clerici, Ghadeer S. Aljuraiban

**Affiliations:** 1 Department of Community Health Sciences, College of Applied Medical Sciences, King Saud University, Turki Alawwal Street, Riyadh 11451, Saudi Arabia; 2 Department of Clinical Laboratory Sciences, College of Applied Medical Sciences, King Saud University, Riyadh 11433, Saudi Arabia; 3 Chair for Biomarkers of Chronic Diseases, Biochemistry Department, College of Science, King Saud University, Riyadh 11451, Saudi Arabia; 4 Department of Pathophysiology and Transplantation, University of Milan, 20122 Milan, Italy; 5 IRCCS Fondazione Don Carlo Gnocchi ONLUS, 20148 Milan, Italy

**Keywords:** gut microbiota, obesity, BMI, %body fat, WHR, microbial alpha diversity, microbial beta diversity

## Abstract

**Simple Summary:**

Obesity is a global health problem associated with increased mortality and morbidity rates. Concurrently, with important advances in therapeutic options for obesity, the global prevalence of obesity has not decreased, and the burden of obesity has become a concerning issue of our times. In addition, the mechanisms underlying this pathology and the etiological factors are incompletely understood. Thus, understanding and clarifying the possible etiological factors for obesity is essential. Over the last decade, researchers have been focusing on the gut microbiota as an element that is implicated in the aetiology of obesity. However, there is limited available epidemiologic evidence in the Middle East, especially in Saudi Arabia. In addition, very little evidence exists elsewhere on the association between the gut microbiota composition and obesity markers in young women of childbearing age. In light of this, we designed a case-control study that explored the gut microbiota composition of Saudi Arabian women with obesity compared with healthy controls using whole-genome shotgun sequencing. Our findings highlight the role of the gut microbiota in obesity and provide significant insight into creating modulation strategies for obesity management through fecal microbiota transfer, antibiotics, probiotics, and prebiotics, offering potential targets for guiding the choice of strain probiotics for gut microbiota modulation in the treatment of obesity.

**Abstract:**

Recent evidence has suggested that the gut microbiota is a possible risk factor for obesity. However, limited evidence is available on the association between the gut microbiota composition and obesity markers in the Middle-Eastern region. We aimed to investigate the association between gut microbiota and obesity markers in a case-control study including 92 Saudi women aged 18–25 years, including participants with obesity (case, *n* = 44) and with normal weight (control, *n* = 48). Anthropometric, body composition, and biochemical data were collected. The whole-genome shotgun technique was used to analyze the gut microbiota. The Shannon alpha and Bray–Curtis beta diversity were determined. The microbial alpha diversity was significantly associated with only the waist-to-hip ratio (WHR) (*p*-value = 0.04), while the microbial beta diversity was significantly associated with body mass index (*p*-value = 0.048), %body fat (*p*-value = 0.018), and WHR (*p*-value = 0.050). Specific bacteria at different taxonomic levels, such as Bacteroidetes and Synergistetes, were positively associated with different obesity markers. Alistipes was higher in the control group compared with the case group. The results highlight the association of the gut microbiota with obesity and suggest that the gut microbiota of Saudi women is associated with specific obesity markers. Future studies are needed to determine the role of the identified strains in the metabolism of individuals with obesity.

## 1. Introduction

Over the last few decades, obesity has been transformed from a relatively minor health issue to a major global threat to public health [1]. Obesity is linked to mortality [1] as well as other chronic health conditions such as diabetes, cardiovascular disease, arthritis, and cancer. According to the World Health Organization global report in 2016, about 13% of the adult population (11% of men and 15% of women) had obesity [2]. In 2016, the age-standardized prevalence of obesity among adults in Saudi Arabia was 35.4%, higher than other neighboring countries such as the United Arab Emirates (31.7%) and Iraq (30.4%), and was more prevalent in women (42.3%) compared with men (30.8%) [3]. As the risk of non-communicable diseases among offspring has been correlated with maternal nutritional status, targeting obesity among women of child-bearing age is pivotal [4].

Although several factors such as unhealthy diet, low physical activity, genetics, age, and disturbed lifestyle have well-established links to obesity [5], exploring other factors related to this major public health issue is of paramount importance. Recently, the gut microbiota has been implicated in the etiology of obesity, and vice versa [6]. The gut microbiota can be defined as all the microorganisms that exist in the gastrointestinal tract, mainly in the colon (bacteria, viruses, protozoa, and fungi), and their collective genetic material [7]. Many factors such as ethnicity, type of diet, physical inactivity, and the use of antibiotics, play an important role in the gut microbiota composition and may induce dysbiosis, which is implicated in the pathogenesis of some diseases, including obesity [7].

Various mechanisms have been proposed from pre-clinical studies to link the composition of the gut microbiota with obesity genesis through, for example, inflammatory and metabolic mechanisms [8]. However, the mechanism behind this association in humans remains unclear [9]. A recent systematic review of observational human studies and clinical trials revealed that the gut microbiota profile of adults with obesity had greater *Firmicutes* and *Fusobacteria*, and lower *Bacteroidetes* and *Lactobacillus paracasei* [10]. Kolida et al. [11] reported that the relative abundance of the major microbial phyla varied significantly between body mass index (BMI) categories. Notably, individuals who had an F:B ≥ 1 were 23% more likely to have higher body weight than those with F:B < 1 [11]. Conversely, another systematic review and meta-analysis of case-control studies reported that the difference in the relative abundance of *Firmicutes* and *Bacteroidetes* in individuals with obesity compared with individuals without obesity was not statistically significant [12]. Such inconsistent findings may be attributed to methodological limitations, such as limited adjustment for potential confounding factors, restricting the generalizability of results, small sample size, restriction to normal-weight participants, and the techniques used to identify the gut microbiota.

Up to date, limited studies have addressed obesity markers, including WHR and %body fat, in relation to the gut microbiota [13,14]. In Saudi Arabia, only three cross-sectional studies have assessed the gut microbiota composition of the Saudi population [15,16,17], with two exclusively in male population samples [15,16]. To the best of our knowledge, no studies have assessed the association between gut microbiota and obesity markers in Saudi women using the gold standard whole-genome shotgun sequencing technique [18]. As the dietary habits and lifestyle of Saudis are different from that of Westerners, identifying the association of the gut microbiota composition with obesity is essential. Therefore, the aim of the present study was to investigate the gut microbiota composition of Saudi Arabian women with obesity compared with healthy controls using whole-genome shotgun sequencing.

## 2. Materials and Methods

### 2.1. Study Design and Setting

The present case-control study was conducted between January 2019 and March 2020 at King Saud University, Riyadh, Saudi Arabia. Saudi female students aged 18–25 years old with obesity (BMI ≥ 30 kg/m^2^) or with normal weight (BMI = 18.5–24.9 kg/m^2^) were invited to participate in the current study through flyers, emails, and word of mouth. Excluded women were those who were pregnant, overweight (BMI = 25.0–29.9 kg/m^2^), those following specific diets (e.g., calorie-restricted diets), with chronic medical conditions especially gastrointestinal diseases (e.g., colon cancer, inflammatory bowel disease, or acute/chronic diarrhea in the previous eight weeks), oncological or endocrine diseases, anorexia, psychiatric disorders, use of multi-vitamins or vitamin B12, or use of antibiotics during the six months prior to stool sample collection. Of the 400 participants assessed, *n* = 105 did not meet inclusion criteria and *n* = 193 did not provide a stool sample. Hence, 102 participants were included in the study, but *n* = 5 participants were excluded because of inadequate DNA sample concentration and *n* = 5 were identified as implausible reporters of energy based on the Goldberg equation [19] and were thus excluded. Thus, a total of 92 Saudi female students were included in the study (48 with normal-weight and 44 with obesity) (Figure S1).

After providing information about the study and obtaining signed consent to participate, along with permission to collect demographic, anthropometric, and biochemical data, including blood and stool samples, each participant was given an appointment to undergo a full assessment at the study clinic. All appointments were booked during the morning period, in which samples were collected at the same time on one day. Participants had the option to withdraw at any stage of the study.

The study protocol was approved by the Institutional Review Board Committee of the Deanship of Scientific Research at King Saud University (IRB #E-19-3625), and all methods were performed in accordance with the Declaration of Helsinki.

### 2.2. Study Tools

#### 2.2.1. Anthropometric Measures

Each measurement was recorded twice, and the average reading was used in the analyses. Weight was recorded to the nearest 0.1 kg with light clothing and without shoes, using an international standard scale (Digital Pearson Scale; ADAM Equipment Inc., Oxford, CT, USA). Height was recorded using the same scale to the nearest 0.5 cm, while standing without shoes and facing the scale.

BMI was calculated by dividing the weight in kilograms by the height in meters squared. Based on BMI levels, participants were divided into cases (BMI of 18.5–24.9 kg/m^2^) and controls (BMI ≥ 30 kg/m^2^) [2].

Waist circumference was measured at the narrowest level between the lowest rib and the umbilicus, and hip circumference was measured at the level of the great trochanter, with legs close together using non-stretchable tape [20]. Both measurements were recorded to the nearest 0.5 cm. If the variation between the measurements was greater than 2 cm, a third measurement was obtained, and the mean of the two closest measurements was used. The WHR was calculated by dividing the mean waist circumference by the mean hip circumference [20]. Participants with WHR of <0.83 were considered as having “normal” WHR, while those with WHR ≥ 0.83 were considered as having a “high” WHR [20]. Muscle mass and %body fat were measured using the bioelectrical impedance device (770 BIA; InBody, Seoul, South Korea) [21]. Normal %body fat was defined as <35% and high %body fat was defined as ≥35% [22].

#### 2.2.2. Clinical Data and Questionnaire

At the clinic, each participant was interviewed by a trained clinical dietitian following the study protocol and using a structured questionnaire consisting of clinical history assessment (participant and family medical history, i.e., diagnosis of obesity or any chronic disease in first- or second-degree relatives), a sociodemographic questionnaire (age, college, family income, occupation, and marital status), the Global Physical Activity Questionnaire (including intensity, duration, frequency, and sitting time) [23], the Pittsburgh Sleep Quality Index questionnaire (sleep quality, sleep latency, sleep duration, sleep efficiency, sleep disturbance, use of sleeping medication, and daytime dysfunction) [24], and the Saudi Food and Drug Authority Food Frequency Questionnaire (SFDA-FFQ) [25]. The SFDA FFQ consists of 10 sections with 133 food items and includes questions regarding the type of cooking, fat used, visible fat consumption, salt, and vitamin intake [25]. Household measures, food modules, and pictures were used to collect information on food consumption and for portion size estimation. Participants were following a regular, balanced diet. The intake of macronutrients was calculated as the percentage of daily energy intake and micronutrients were calculated per 1000 kcal. The SFDA-FFQ was analyzed using a Microsoft Excel sheet based on the 1996 Saudi food composition table and the seventh edition of McCance and Widdowson’s Composition of Foods Integrated Dataset [26].

#### 2.2.3. Stool Sample and DNA Extraction

Fecal samples were collected under aseptic conditions in clean, dry, screw-top containers. Once the samples were collected, they were transported in a box with dried ice to the study laboratory at King Saud University, where they were stored at −80 °C for further analysis. The DNA was extracted from 0.25 g of frozen stool aliquots using the QIAamp PowerFecal DNA Isolation Kit (Qiagen, Hilden, Germany). The DNA was eluted in 100 μL of the C6 elution buffer according to the kit protocol. The purity (260/280 ratio) and concentration of the extracted DNA were measured using a NanoDrop spectrophotometer (NanoDrop Technologies, Wilmington, Delaware, USA). The extracted DNA samples were stored at −20 °C. DNA samples were shipped through the World Courier company in a box with dried ice to the CosmosID company (CosmosID Inc., Rockville, MD, USA) for library preparation, sequencing, and identification of the gut microbiota.

### 2.3. Bioinformatics and Statistical Analysis

DNA libraries were prepared using the Illumina Nextera XT Library Preparation Kit (Illumina, San Diego, CA, USA) according to a modified protocol. A Qubit^®^ fluorimeter (Thermo Fisher, Milan, Italy) was used for quantitative assessment. After library preparation, and samples (2 × 150 bp) were sequenced using an Illumina sequencer.

Determination of the gut microbiota composition at the level of the major microbial phyla was done by identifying the total bacterial DNA and *Bacteroidetes* and *Firmicutes* DNA using the whole-genome shotgun sequencing. Unassembled sequencing reads were directly analyzed for multi-kingdom microbiome analysis, profiling of antibiotic resistance and virulence genes, and quantification of the relative abundance of organisms using the CosmosID bioinformatics platform (CosmosID Inc., Rockville, MD, USA) [18].

The normality of all quantitative variables was tested before the analysis. Descriptive analysis results are presented as frequencies and percentages for categorical data and as means and standard deviations for continuous data. Pearson’s chi-squared test was used to determine the associations between the categorical variables and the outcomes, while the independent samples t-test was used for the continuous variables and outcomes.

Alpha diversity, used to measure the richness of the gut microbiota, and beta diversity, used to measure its composition, were calculated from the species level relative abundance matrices from CosmosID taxonomic analysis using the R software Vegan package (Version 2.5-6). Alpha and beta analyses were done for cases and controls, for those with normal and high WHR, and those with normal and high %body fat. Wilcoxon Rank-Sum tests were done to investigate the statistical difference in the alpha diversity of the gut microbiota based on the Shannon index between the cases and controls groups using the ggsignif package for R. Boxplots were generated using the same package for R [27].

The statistical significance of the beta diversity between cases and controls group was determined with the nonparametric PERMANOVA analysis based on the Bray–Curtis distance using Vegan’s function adonis2. The principal coordinate analysis plot was generated using the Vegan package’s PCoA function. PC1 represents the first dimension, PC2 the second dimension, and PC3 the third dimension. Plots were visualized using the ggpubr package for R [27,28].

Linear Discriminant Analysis Effect Size (LEfSe) figures were generated using the LEfSe tool from the Huttenhower lab, based on the phylum, genus, species, strain, and functional matrices from the CosmosID analysis. In the LEfSe figures, red bars to the right convey that the feature in that group is more abundant in the case group than in the control group. Blue bars to the left convey that the organism is more abundant in the control group. Linear regression analysis was done to identify associations between the gut microbiota at various taxonomic levels and obesity markers.

Statistical analysis was performed using IBM SPSS Statistics for Windows (version 24; IBM Corp., Armonk, NY, USA). A *p*-value of <0.05 was used to indicate statistical significance.

## 3. Results

### 3.1. Descriptive Characteristics

A total of 92 Saudi female college students were included in the current study. The descriptive characteristics of the participants are presented in Table 1. Participants in the case group had a higher waist, hip, %body fat, total body protein, skeletal muscle mass, muscle mass (*p*-value < 0.001), and WHR (*p*-value = 0.01) compared with those in the control group. Participants in the case group had higher total cholesterol (*p*-value = 0.01), fasting blood glucose (*p*-value = 0.04), low-density lipoprotein cholesterol (*p*-value = 0.01), total cholesterol/high-density lipoprotein cholesterol ratio (*p*-value = 0.05), triglyceride, insulin, homeostatic model assessment for insulin resistance, and homeostasis model assessment of β-cell function (*p*-value < 0.001). Furthermore, no significant differences in daily intake of energy (*p*-value = 0.08), fat% (*p*-value = 0.89), carbohydrate% (*p*-value = 0.91), and protein% were observed between the case and control group (Table S1). Compared with those in the case group, participants had slightly lower median of total MET-minutes [540.0 (260.0–1680.0)] compared with the control group [714.0 (300.0–1200.0)]; however, the difference was not statistically significant (*p*-value = 0.96) (Table S1).

### 3.2. Alpha Diversity Analysis

**Figure 1:**
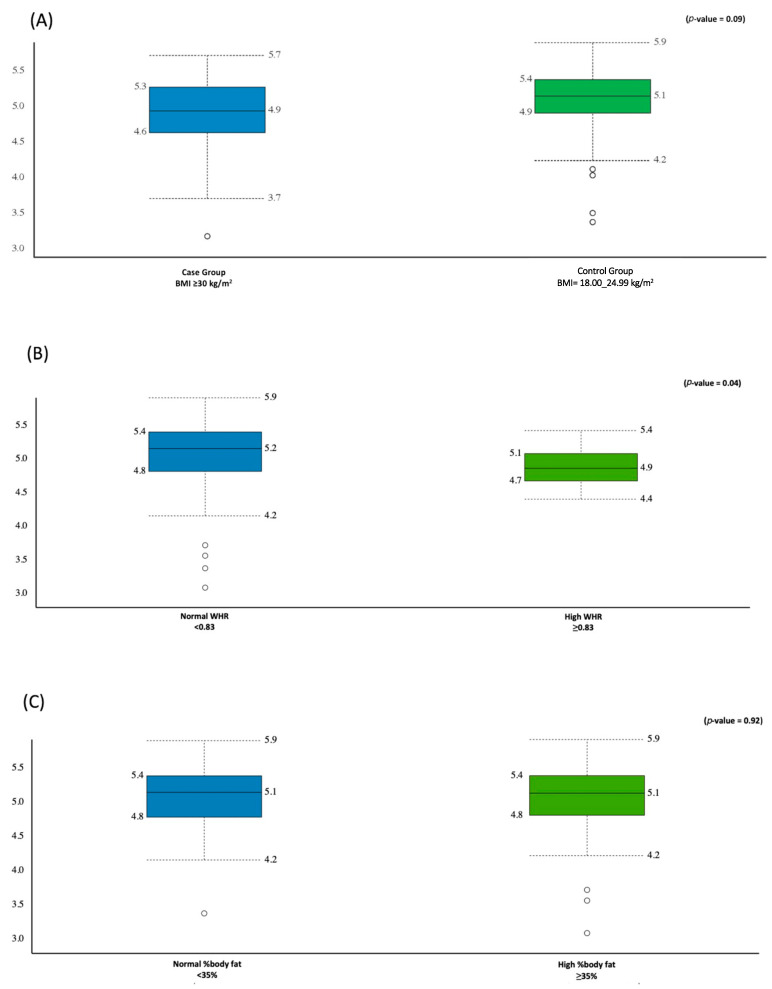
Comparison between the gut microbiota alpha diversity (Shannon–Wiener index) and obesity markers: (**A**) relative to the BMI, (**B**) relative to WHR, (**C**) relative to %body fat.

A total of 62.521 M sequencing reads were obtained from the 92 fecal samples, with a mean value of 5.705 M ± 1.768 M reads generated per participant. The Shannon index was used as an indicator of the microbial diversity and was higher in the control group compared with the case group (5.1 ± 0.5 vs. 4.9 ± 0.5); however, this difference was not statistically significant (*p*-value = 0.09) (Figure 1A).

The Shannon index was higher in the group with normal WHR than the group with high WHR (5.2 ± 0.5, 4.9 ± 0.3, *p*-value = 0.04), respectively (Figure 1B). There was no difference in the microbial diversity between the group with high and the group with normal %body fat (≈ 5.1 ± 0.5, *p*-value = 0.92) (Figure 1C).

### 3.3. Beta Diversity Analysis

**Figure 2:**
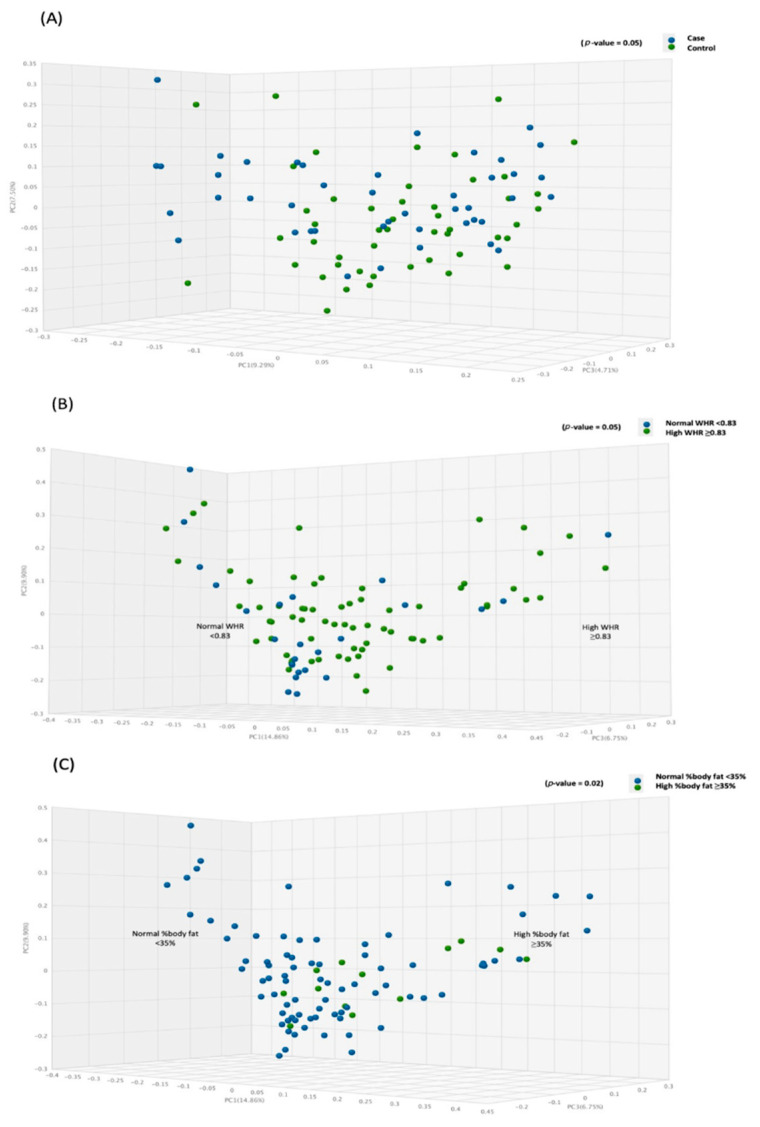
Comparison between gut microbiota beta diversity (PCoA) and obesity markers: (**A**) relative to the BMI, (**B**) relative to WHR, and (**C**) relative to %body fat.

The principal coordinate analysis was performed using the paired Bray–Curtis similarity index to observe the difference in the beta diversity between the cases and the controls groups of different obesity markers. The results are demonstrated by PC1, PC2, and PC3, accounting for 14.9, 9.9, and 6.8% of the total variations. Figure 2 shows that the microbial beta diversity was significant between the case and control groups (*p*-value = 0.048) (Figure 2A), high and normal WHR (*p*-value = 0.050) (Figure 2B), and high and normal %body fat (*p*-value = 0.018) (Figure 2C).

### 3.4. Gut Microbiota Signatures in the Case and Control Groups

**Figure 3:**
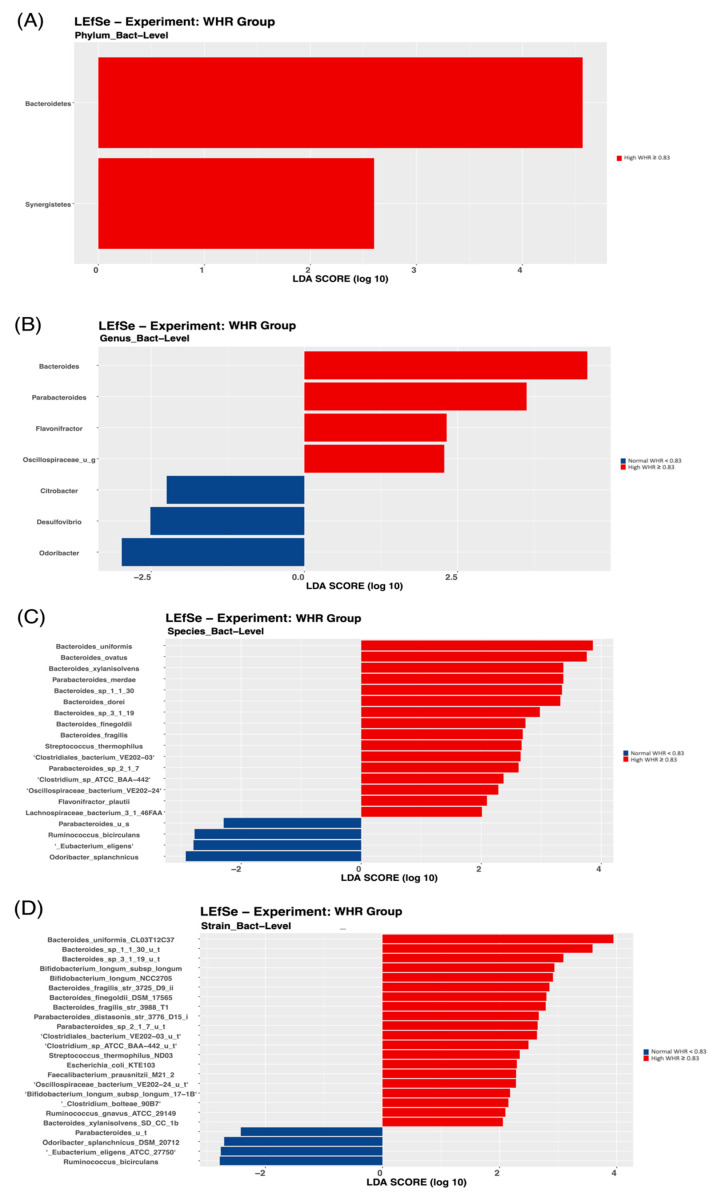
Linear Discriminant Analysis Effect Size (LEfSe) of microbial taxa between the group with high WHR and the group with normal WHR: (**A**) phylum level, (**B**) genus level, (**C**) species level, and (**D**) strain level.

To further identify the bacterial taxonomic groups, we performed a biomarker analysis using LEfSe. The results showed that at the phylum level, only the *Bacteroidetes* and *Synergistetes* phyla were most discriminative of the participants with a high WHR (Figure 3A).

**Figure 4:**
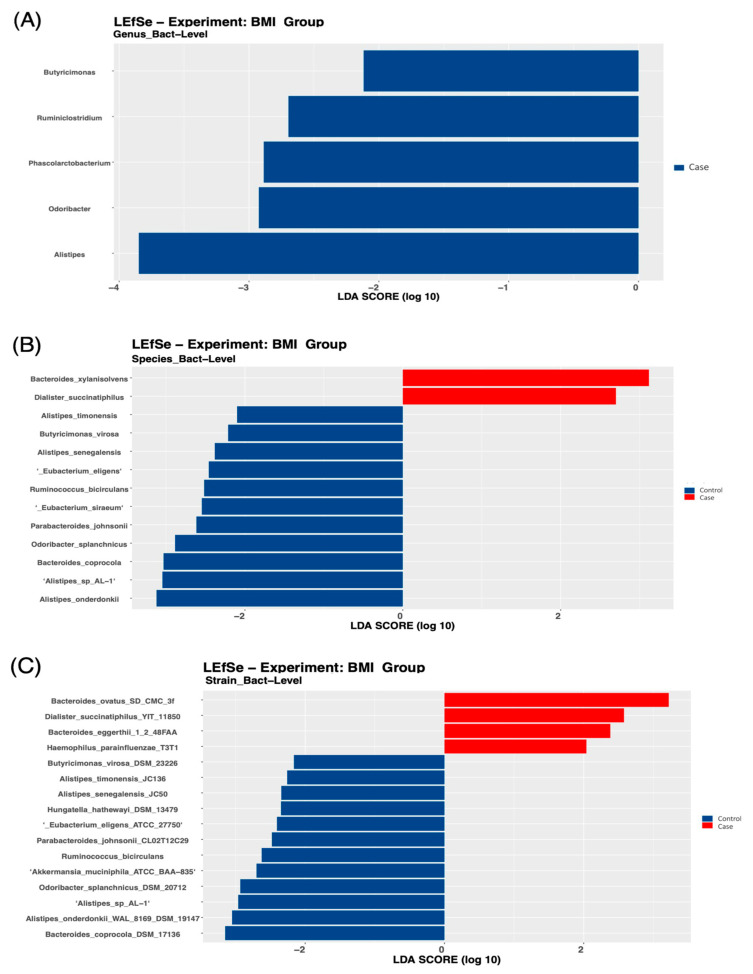
Linear Discriminant Analysis Effect Size (LEfSe) of the microbial taxa between the group with obesity (case group) and the group with normal BMI (control group): (**A**) genus level, (**B**) species level, and (**C**) strain level.

**Figure 5:**
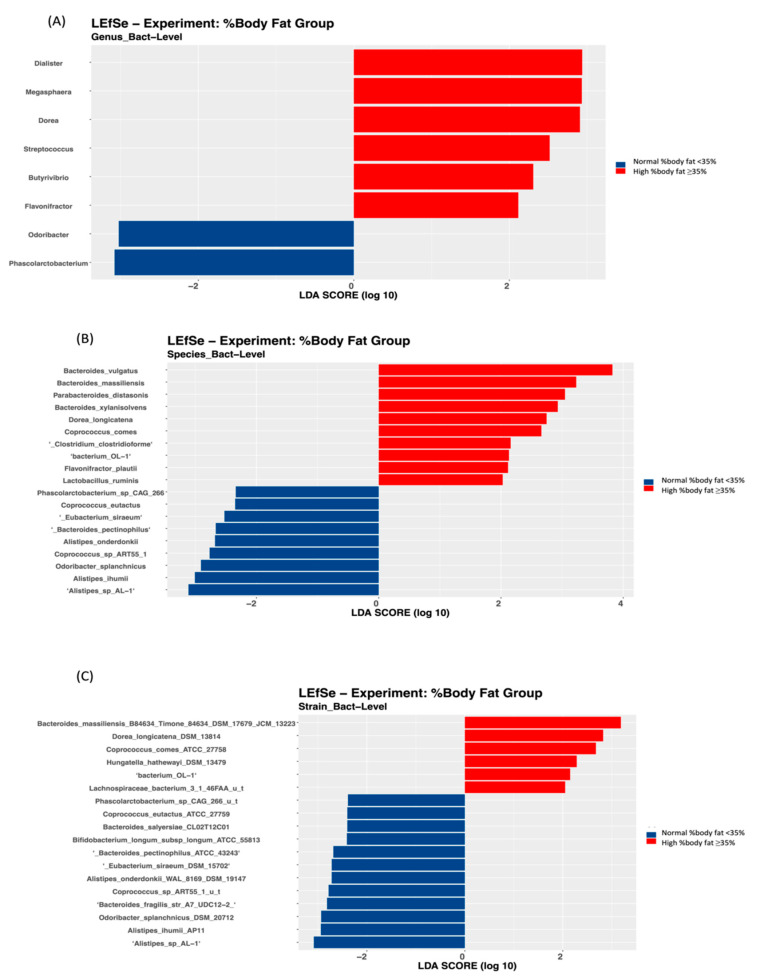
Linear Discriminant Analysis Effect Size (LEfSe) of microbial taxa between the group with high %body fat and the group with normal %body fat: (**A**) genus level, (**B**) species level, and (**C**) strain level.

At the genus level, *Alistipes* was higher in the control group compared with the case group (Figure 4A), *Bacteroides* was higher in the high compared with the normal WHR group (Figure 3B), and *Flavonifractor* was enriched in those with a high WHR and %body fat (Figure 3B and Figure 5A).

Moreover, different species were significantly discriminative of the case group, and in those with high WHR and %body fat. For example, two species were found in the case group (Figure 4B), 15 species in those with high WHR (Figure 3C), and 12 species, such as *Dorea Longicatena*, in those with high %body fat (Figure 5B). *Bacteroides Xylanisolvens* was considerably enriched in the case group (Figure 4B) and in those with high WHR (Figure 3C) and high %body fat (Figure 5B). At the strain level, four strains were significantly discriminative of the case group (Figure 4C), 21 in those with high WHR (Figure 3D) and 7 in those with high %body fat (Figure 5C).

**Figure 6:**
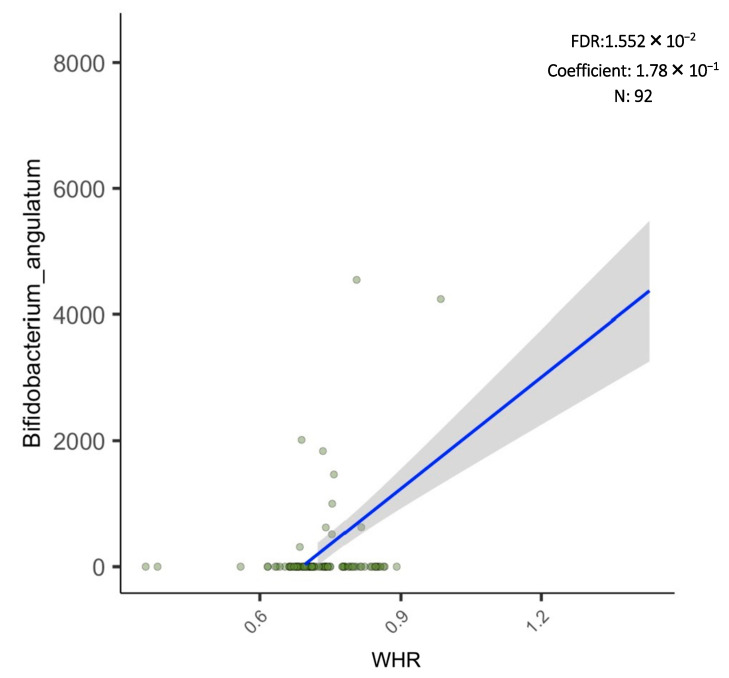
Linear regression analysis of *Bifidobacterium angulatum* and WHR.

**Figure 7:**
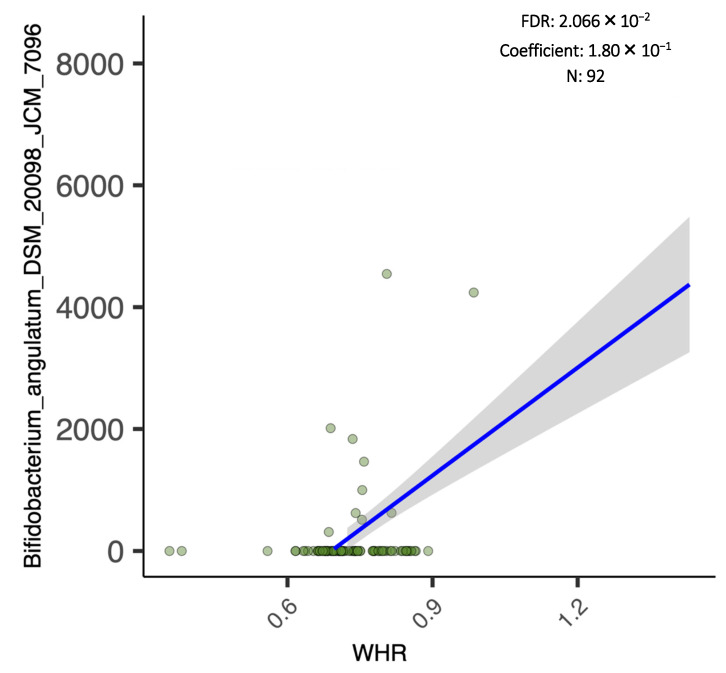
Linear regression analysis of *Bifidobacterium angulatum DSM 20098*, *JCM 7096* and WHR.

Additionally, the results of the regression analysis revealed that the abundance score of *Bifidobacterium Angulatum* species and *Bifidobacterium Angulatum DSM 20098*, *JCM 7096* strain were significantly and positively associated with the numeric WHR (Figure 6 and Figure 7).

## 4. Discussion

Studies investigating the differences in gut microbiota composition between normal-weight and individuals with obesity, especially in the Middle Eastern region, are limited. Our findings contribute to the growing evidence that obesity markers are associated with beta diversity of the gut microbiota. Furthermore, WHR was significantly associated with alpha diversity of the gut microbiota. The findings revealed that the specific phylum, species, genera, and strains of the gut microbiota of Saudi Arabian women were associated with specific obesity markers.

The findings of the current study are comparable with those reported by Palmas et al., with a significant difference in beta diversity (composition) between the group with obesity and with normal-weight (*p*-value = 0.002) [13], highlighting the variation in the microbial communities between study cohorts. Previous reports including population samples from Saudi Arabia demonstrated a significant association between specific bacteria and BMI; however, these studies did not reveal an association between beta diversity of the gut microbiota and BMI in men [15], or in men and women [17]. Inconsistent findings may be attributed to sex, as functional gene richness of the colon may be associated with sex hormones, and could play a role in regulating the gut microbiota composition [29]. Another key point is that Saudi Arabia is a large country with five geographical divisions, each with distinct dietary and lifestyle habits [30]. There is evidence that gut microbiota differs not only between different countries, but also within the same country and between different localities, because of the geographical and socio-economic differences [31]. In addition, the young age of participants in our study may also explain the inconsistent results, as gut microbiota composition differs among various age groups [32]. Another factor to consider is the different techniques used for the identification of the gut microbiota; in the current study, we used the gold-standard whole-genome shotgun, while previous studies used 16S rRNA sequencing with MiSeq technology [15,17].

Interestingly, we observed that participants with a high BMI had a specific gut microbiota profile compared with those with a normal BMI. Similarly, Yasir et al. [15] revealed that Saudi men with a high BMI had a higher abundance of *Firmicutes* and *Dorea* compared with those with a normal BMI (*p*-value < 0.01), suggesting the role of the gut microbiota in obesity development. We also found that BMI was not associated with the alpha diversity (richness) of the gut microbiota, comparable to previous studies done on Saudi population samples [15,17], but inconsistent with most studies done elsewhere [11,33]. As noted in previous studies, the differences in gut microbiota composition may be attributed to varying lifestyle and dietary factors. For example, Yaser et al. [15] reported that the composition of the gut microbiota of a sample population from Saudi Arabia was different compared with a sample population from France, even when comparing the same BMI categories, further emphasizing that gut microbiota composition is influenced by cultural and dietary factors.

The current study is the first to investigate other obesity markers such as %body fat and WHR in relation to the gut microbiota in the Middle Eastern region. We reported that WHR was significantly associated with both beta and alpha diversity. Additionally, individuals with a high WHR had a different gut microbiota composition compared with those with a normal WHR. These findings are comparable to a recent report by Gwendolyn et al. [14], where the alpha diversity of the gut microbiota was significantly associated with upper-mid arm circumference, waist circumference, and WHR. Furthermore, we found that %body fat was significantly associated with the beta diversity. Participants with high %body fat had specific gut microbiota profiles compared with those with normal %body fat. In contrast, no significant differences were found between %body fat and alpha diversity (richness) of the gut microbiota. Notably, most of our participants in the case and control groups had high %body fat (mean 42.56%±9.34%) [34]. Comparably, a recent multi-center randomized controlled trial across eight countries revealed that the gut microbiota composition was significantly affected by %body fat after a low-energy diet intervention (*p* < 0.001) [35]. Specifically, the baseline gut microbiota was a strong predictor of the change in the total %body fat during the energy restriction period [35].

The results of the gut microbiota signatures in our study demonstrated that *Bacteroidetes* and *Synergistetes* phyla were the most discriminative of participants with a high WHR. A recent systematic review and meta-analysis revealed that the abundance of *Bacteroidetes* in individuals with obesity compared with those with normal weight showed contradictory results [36]. Although no specific phyla were identified in the case group compared with the control group and in those with high %body fat compared with normal %body fat, bacteria at different taxonomic levels were significantly discriminative of the case group and those with a high WHR and %body. Consistent with our findings, a previous study revealed that Korean twins demonstrated strain-level differences in composition, despite species-level similarities [37]. This supports evidence that health-related microbiome studies should extend to more complex communities, not just to the higher taxonomic levels, as different strains of the same species could vary widely in their relation to disease.

At the genus level, the abundance of *Alistipes* was higher in the control group, while the abundance of *Bacteroides* was higher in those with a high WHR. *Alistipes* has been identified as a new genus that is significantly increased in high-fat-diet-fed mice compared with controls [38]. A previous systematic review revealed that individuals with obesity had a higher abundance of *Alistipes* and a lower abundance of *Bacteroides* [10]. Controversial findings indicate that Saudi Arabian women may have a unique composition of the gut microbiota, attributed to varying lifestyle and dietary factors. In Saudi Arabia, there is a current shift from traditional Saudi dietary patterns to Westernized dietary habits, namely diets that are energy dense, and high in animal protein, total and saturated fats, and simple sugars [39].

We reported that *Bacteroides Xylanisolvens* was high in the case group and in those with high WHR and %body compared with their counterparts. Inconsistent with these findings, a recent report revealed that *Bacteroides Xylanisolvens* was significantly associated with obesity in a sample population from China [40]. Nevertheless, the role of *Bacteroides Xylanisolvens* in obesity development has not been fully identified in the literature and future studies are warranted. We also found that *Dorea longicatena* was high in those with high %body fat, comparable with a previous study conducted on Saudi men with a high BMI level who were found to have a higher abundance of *Dorea* compared with those with a normal BMI (*p*-value = 0.004) [15]. The hgher abundance of the *Dorea* genus was reportedly associated with increased intestinal permeability [41].

One of the interesting findings of the current study is that *Bifidobacterium Angulatum* species were significantly and positively associated with WHR. It has been suggested that different strains of *Bifidobacterium* may lead to different responses in fat distribution and metabolism [42]. This premise may have been the case in one of the few probiotic randomized clinical trials done in Saudi Arabia among patients with T2DM and obesity [43], where multi-strain probiotics supplementation that included *Bifidobacterium bifidum* and *Bifidobacterium lactis* led to a clinically significant decrease in WHR in favor of the probiotic group after 6 months of intervention in both intent-to-treat [placebo 0.0% vs. probiotics 1.11%; *p* = 0.02] and per-protocol analysis [placebo 0.0% vs. probiotics 1.11%; *p* = 0.01)] [43]. However, this significant decrease in visceral adiposity, as seen in WHR, was not observed after 3 months [44]. Therefore, the identification of strains present in the gut microbiota composition and its association with measures of adiposity in high risk populations may serve to enhance the success of these interventions.

To the best of our knowledge, the current study is the first to examine the association between the gut microbiota and obesity markers, including WHR and %body fat, among women at different taxonomic levels. We used the whole-genome shotgun technique; the gold-standard for taxonomic identification at the species level, with a higher sensitivity and resolution compared with other techniques. Furthermore, extensive measures were applied to increase the accuracy of the data collection process, such as in-person interviews and the use of validated questionnaires [23,24,25] and measurement tools. We applied an innovative combination of tools to measure microbial diversity in the multivariate data—one for assessing the richness of the gut microbiota, and another for assessing the variation in its composition. However, the study limitations should be addressed. These include the observational design, which does not permit causation and measurement error; although we used the average of measurements to increase precision, there was the potential of recall bias and under-reporting of energy during data collection [45], and the inclusion of only female participants at a single setting. Moreover, the number of reads per sample was variable and may have affected the identification of bacteria with less than 0.1% abundance. However, when we converted data to the relative abundance, we normalized by library size to account for read depth and to allow for comparison among the study sample, regardless of read depth. Various studies have shown that the relative proportions of read assignments stayed relatively consistent, regardless of depth [46,47,48].

## 5. Conclusions

In conclusion, the present case-control study revealed, for the first time, differences in the gut microbiota composition of Arab women with and without obesity. Beta diversity (composition) was associated with most obesity indices, while alpha diversity (richness) was associated only with WHR. These observational findings suggest that microbiota composition is associated with obesity, and support the growing evidence that gut microbial diversity is related to adiposity indices, independent of the compositional variations across geographically distinct populations. The associations and differences elicited may be explained by a plethora of factors, including differences in dietary intake, all of which can only be verified using a longitudinal approach. The present hypothesis-generating study warrants investigations focusing on interventions that can favorably alter the gut microbiota of populations at risk, taking into consideration the role of identified strains associated with adiposity measures.

## Figures and Tables

**Table 1:** General characteristics of the studied population ^1^. Body mass index (BMI), waist-to-hip ratio (WHR), fasting blood glucose (FBG), high-density lipoprotein (HDL), low-density lipoprotein (LDL), Homeostatic Model Assessment for Insulin Resistance (HOMA-IR), and the homeostasis model assessment of β-cell function (HOMA-β).

Characteristics	Total (*n* = 92)	Control Group BMI = 18.50–24.99 (*n* = 48)	Case Group BMI ≥ 30.00 (*n* = 44)	*p*-Value
Age (Years)	21.1 ± 1.5	20.6 ± 1.1	21.6 ± 1.7	<0.01
Age of Menarche	12.4 ± 1.0	12.5 ±1.0	12.3 ± 1.1	0.31
Anthropometric measurements				
Height (cm)	157.7 ± 5.2	158.0 ± 5.7	157.3 ± 4.5	0.48
Weight (kg)	70.7 ± 19.7	54.3 ± 6.0	89.1 ± 11.8	<0.001
BMI (kg/m^2^)	28.6 ± 8.0	21.7 ± 1.9	36.0 ± 4.7	<0.001
Waist (cm)	80.4 ± 17.4	67.7 ± 4.3	94.5 ± 15.4	<0.001
Hip (cm)	109.2 ± 16.4	96.4 ± 7.6	123.5 ± 10.8	<0.001
WHR (ratio)	0.7 ± 0.1	0.7 ± 0.1	0.8 ± 0.1	0.01
Fat (%)	42.5 ± 9.4	34.8 ± 5.5	51.1 ± 3.3	<0.001
Total body protein (kg)	7.6 ± 1.1	6.9 ± 0.7	8.5 ± 0.9	<0.001
Skeletal muscle mass (kg)	21.0 ± 3.5	18.6 ± 2.2	23.6 ± 2.6	<0.001
Muscle mass (%)	28.2 ± 7.0	29.6 ± 9.3	26.6 ± 1.9	0.04
Total Body Water (L)	28.7 ± 4.3	25.8 ± 2.7	31.9 ± 3.3	<0.001
Fluid (%)	42.1 ± 6.8	47.7 ± 4.0	35.9 ± 2.3	<0.001
Biochemical data				
Total cholesterol (mmol/L)	4.1 ± 1.5	3.6 ± 1.7	4.5 ± 1.0	0.01
FBG (mmol/L)	4.6 ± 0.7	4.5 ± 0.8	4.8 ± 0.6	0.04
HDL-cholesterol (mmol/L)	1.0 ± 0.3	0.9 ± 0.4	1.0 ± 0.3	0.24
LDL-cholesterol (mmol/L)	2.9 ± 1.3	2.6 ± 1.5	3.3 ± 1.0	0.01
Total cholesterol/HDL ratio	4.3 ± 1.7	3.9 ± 1.7	4.7 ± 1.7	0.05
Triglyceride (mmol/L) #	0.7 (0.5–1.0)	0.5 (0.4–0.7)	1.0 (0.8–1.1)	<0.001
Insulin (µIU/mL) #	9.9 (6.0–15.8)	6.4 (5.0–9.3)	15.2 (12.2–20.3)	<0.001
HOMA-IR #	2.0 (1.1–3.6)	1.2 (0.9–1.6)	3.5 (2.3–4.4)	<0.001
HOMA-β #	165.7 (112.0–288.1)	114.5 (71.8–148.3)	224.5 (182.7–316.1)	<0.001

^1^ Variables are presented as (mean ± standard deviation (SD)) or *n* (%). Non-normal variables presented as median (1st quartile–3rd quartile). # Indicates non-normal variables.

## Data Availability

The datasets generated for this study can be found in the Figshare repository: 10.6084/m9.figshare.20106176.

## Supplementary Materials

The following supporting information can be downloaded at: https://www.mdpi.com/article/10.3390/biology11111586/s1. Figure S1: Flow chart of participation in the study; Table S1: General characteristics of the studied population.
