# Supplementary material for: Influence of Adiposity on the Gut Microbiota Composition of Arab Women: A Case-Control Study
Source: Biology (Basel). 2022 Oct 28;11(11):1586. doi: 10.3390/biology11111586 (PMC9687783; doi:10.3390/biology11111586)
Supplement: Supplementary file 1 [file biology-11-01586-s001.zip › Table S1.pdf]

Table S1. General characteristics of the studied population[1]

Characteristics	Total (n=92)	Control group BMI = 18.50-24.99 (n=48)	Case group BMI ≥30.00 (n=44)	P-value
Physical Activity				
Sitting (Min\day)	420.0 (300.0 - 600.0)	420.0 (300.0 - 600.0)	360.0 (300.0 - 600.0)	0.67
Total MET\week	620.0 (300.0 - 1350.0)	714.0 (300.0 - 1200.0)	540.0 (260.0 - 1680.0)	0.96
PSS Categories				
Total PSS score	18.9 ± 6.4	19.3 ± 6.0	18.5 ± 6.5	0.33
Sleep parameters				
Total hours of sleep	5.0 ± 1.9	5.0 ± 1.8	4.9 ± 2.1	0.73
PSQI total	8.1 ± 2.8	7.4 ± 2.8	8.9 ± 2.6	0.01
Diet History				
Energy (Kcal/day)	3376.9 (2658.3 - 4412.4)	3613.2 (2881.4 - 4587.7)	3060.3 (2351.0 - 4184.2)	0.08
Fat (%of total kcal)	38.3 (33.3 - 45.0)	37.6 (33.6 - 43.9)	39.5 (32.9 - 45.5)	0.89
Protein (%of total kcal)	13.5 (11.8 - 15.6)	13.3 (12.2 - 15.4)	13.5 (11.5 - 16.0)	0.91
CHO (%of total kcal)	46.5 (40.6 - 52.9)	47.4 (40.4 - 52.6)	44.4 (40.8 - 55.2)	0.91
Fiber (g/1000kcal)	9.7 (7.9 - 11.6)	9.9 (8.0 - 12.0)	9.7 (7.7 - 11.3)	0.30

[1] Variables are presented as (mean ± standard deviation (SD)) or n (%). Non-normal variables presented as median (1st quartile – 3rd quartile)

Perceived stress scale (PSS), The Pittsburgh Sleep Quality Index (PSQI), Carbohydrate (CHO)

All the micronutrients were calculated per 1000 kcal
