# Supplementary figures and images for: Influence of Adiposity on the Gut Microbiota Composition of Arab Women: A Case-Control Study
Source: Biology (Basel). 2022 Oct 28;11(11):1586. doi: 10.3390/biology11111586 (PMC9687783; doi:10.3390/biology11111586)

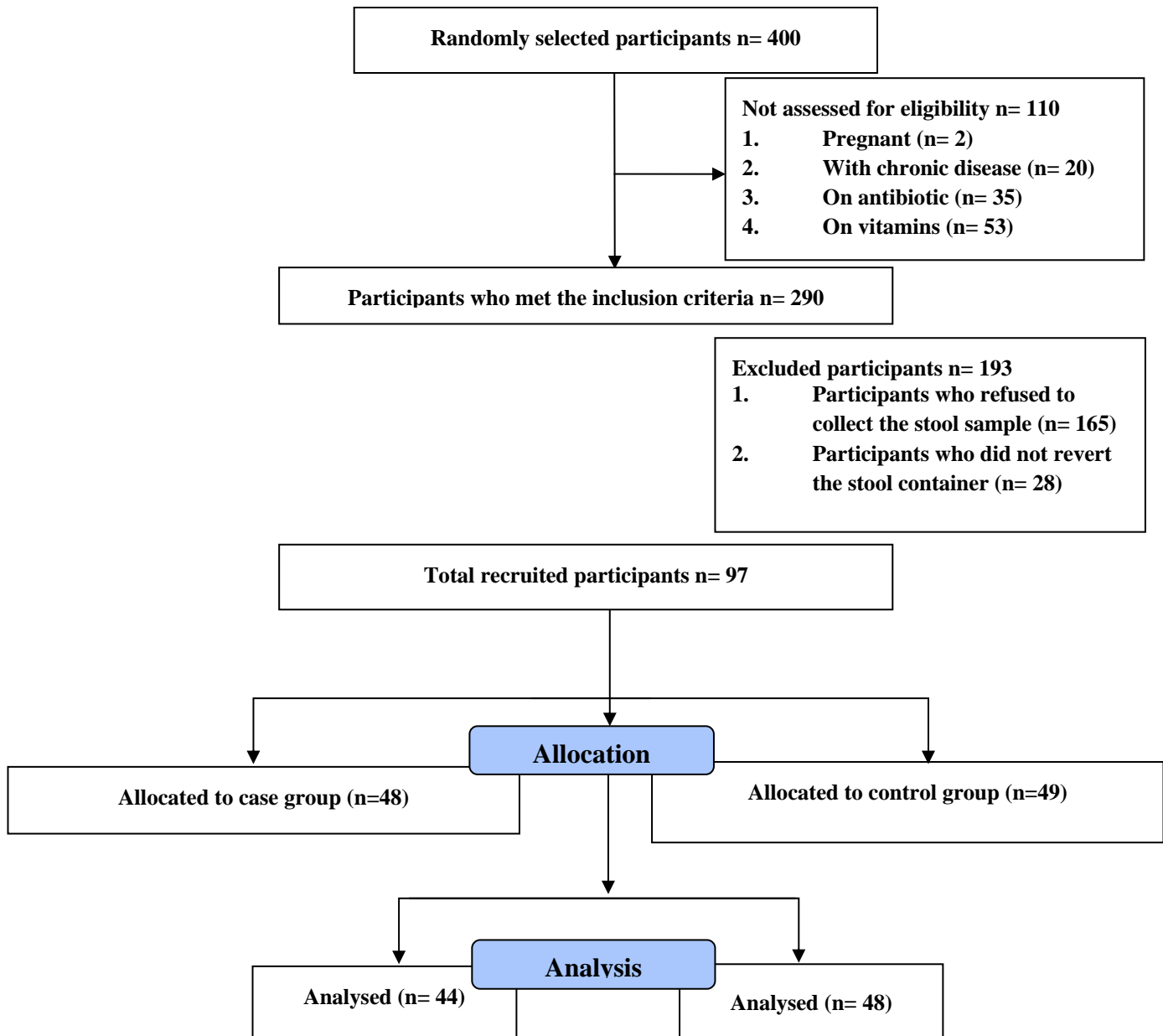

**Figure S1.** Flow chart of participation in the study

Supplement: Supplementary file 1 [file biology-11-01586-s001.zip › Figure S1.pdf]
